# Adult-Onset Episodic Rhabdomyolysis in a Patient With a Heterozygous Lipin 1 (LPIN1) Mutation: A Case Report

**DOI:** 10.7759/cureus.76772

**Published:** 2025-01-01

**Authors:** Naman Bareja, Rafail A Chionatos, Camelia Valhuerdi Porto, Nikita Srinivasan, Mehdi Ghasemi

**Affiliations:** 1 Neurology, Tufts Medical Center, Boston, USA; 2 Neurology, Lahey Hospital and Medical Center, Burlington, USA

**Keywords:** hyperckemia, lipin-1, lpin1 mutation, peripheral muscle weakness, rhabdomyolysis

## Abstract

Lipin-1 (*LPIN1*) is essential in lipid metabolism, with mutations commonly causing severe, recurrent rhabdomyolysis, especially in children. Here, we present a rare case of adult-onset myopathy and rhabdomyolysis in a 48-year-old male firefighter, a heterozygous carrier of an *LPIN1* exon 18 deletion. The patient experienced fatigue, muscle loss, and exercise intolerance over two to three years. Initial evaluations revealed mildly elevated creatine kinase (CK) levels (297 U/L), with unremarkable results from further tests, including electromyography, antinuclear antibodies, myasthenia gravis antibody, and myositis antibody panels. Six months later, he had worsening muscle stiffness, pain, and darkened urine. In the emergency department, his CK was 4000 U/L, with elevated aldolase and transaminases. Hospitalization and hydration treatment normalized his CK levels. Genetic testing through a Comprehensive Neuromuscular Disorder Panel identified a heterozygous *LPIN1* exon 18 deletion. To our knowledge, this case is significant as the first reported instance of adult-onset myopathy and rhabdomyolysis in a heterozygous *LPIN1* carrier without statin exposure. While *LPIN1* mutations typically cause pediatric-onset rhabdomyolysis, this case highlights the need to consider *LPIN1* mutations in adults with episodic myopathy and rhabdomyolysis when other causes are ruled out. Genetic testing is crucial for diagnosis and management.

## Introduction

Lipin-1 (*LPIN1*) is an important protein comprising 890 amino acids and is predominantly expressed in skeletal muscle, adipose tissue, liver, and myocardium [1]. The *LPIN1 *gene is located on chromosome 2p25.1 and encodes the enzyme *LPIN1*. This enzyme plays a pivotal role in lipid metabolism by converting phosphatidic acid to diacylglycerol, an essential precursor for both triglycerides and phospholipids [2]. Mutations in the *LPIN1 *gene are recognized as one of the most common causes of recurrent rhabdomyolysis in the pediatric population [3]. Typically, patients with homozygous or compound heterozygous *LPIN1* mutations experience episodes of severe rhabdomyolysis (creatine kinase (CK) levels exceeding 10,000 U/L), often precipitated by triggers such as febrile illness, intense physical exercise, or fasting. Notably, the onset of these episodes generally occurs before the age of six, indicating an early manifestation of the disorder [3,4]. Despite the severity of these episodes, patients are usually asymptomatic between occurrences, suggesting that the condition is episodic in nature.

It is noteworthy that up to 40% of individuals who are heterozygous carriers of *LPIN1 *mutations may exhibit symptoms such as muscle cramps, myalgias, and exercise-induced muscle pain [4]. Additionally, statin-induced myopathy has been documented in a heterozygous carrier [5]. We present a unique case that adds to the complexity of *LPIN1*-related disorders. This case involves an adult-onset recurrent rhabdomyolysis in a heterozygous patient who had no prior exposure to statins. This observation is particularly noteworthy as it suggests that even in the absence of common triggers like statin use, heterozygous *LPIN1* mutations can lead to significant muscle pathology later in life. This case highlights the variability in clinical presentation and the need for further research to understand the underlying mechanisms and potential risk factors associated with *LPIN1 *mutations.

## Case presentation

A 48-year-old male firefighter sought medical advice initially from his primary care physician in June 2023 due to a three-year history of fatigue, exercise intolerance, and muscle mass loss in the upper extremities. Despite previously being able to lift over 200 pounds on the bench press, this capability had decreased to 135 pounds over this time. He did not experience any difficulty swallowing, shortness of breath, double vision, or weakness in the lower extremities. Despite adhering to a strict strength training regimen, his symptoms first became noticeable during a fire response, where moderate exertion resulted in significant fatigue, particularly in his lower limbs. Of note, the patient did not recall any episodes indicative of rhabdomyolysis or instances of intermittent or persistent muscle weakness or fatigue during childhood. An initial investigation revealed a slightly elevated CK level of 297 U/L (normal range: 46-171 U/L), while his complete blood count (CBC) with differential, basic metabolic panel (BMP), thyroid-stimulating hormone (TSH), and liver function tests (LFT) were unremarkable (Table 1).

**Table 1:** The results of blood testing in the presented case with muscle fatigue and rhabdomyolysis. ^*^Myositis panel include SAE1 (SUMO activating enzyme), NXP2 (Nuclear matrix protein-2), MDA5 (CADM-140), TIF-1 gamma (155 kDa), Mi-2 (nuclear helicase protein), P155/140, PL-12 (alanyl-tRNA synthetase), PL-7 (threonyl-tRNA synthetase), OJ (isoleucyl-tRNA synthetase), EJ (glycyl-tRNA synthetase), SRP (Signal Recognition Particle), Jo-1 (Histidyl-tRNA Synthetase), Ku, Smith/RNP (ENA), PM/Scl 100, SSA-52 (Ro52), SSA-60 (Ro60), Fibrillarin (U3 RNP) antibodies BUN: blood urea nitrogen; AST: aspartate aminotransferase; ALT: alanine aminotransferase; CK: creatine kinase; TSH: thyroid-stimulating hormone; ESR: erythrocyte sedimentation rate; CRP: C-reactive protein; GAA: acid alpha-glucosidase

Measurements	Results				Reference Range
	June 2023	August 2023	February 2024	March 2024	
WBC count	4.97	4.97	4.42	-	4.00-10.8 K/uL
RBC count	4.82	4.82	4.5	-	4.70-6.10 M/uL
Platelet count	220	220	204	-	150-400 K/uL
Sodium	137	138	141	-	134-145 mmol/L
Potassium	4	4.2	4.1	-	3.5-5.1 mmol/L
Chloride	103	103	106	-	98-107 mmol/L
BUN	13	21	20	-	9-23 mg/dL
Creatinine	0.96	0.96	0.99	-	0.6-1.10 mg/dL
Glucose	94	97	92	-	74-100 mg/dL
Calcium	9.2	8.9	8.5	-	8.3-10.6 mg/dL
Magnesium	-	-	1.8	-	1.6-2.6 mg/dL
Phosphate	-	-	3.1	-	2.3-4.6 mg/dL
AST	30	-	59	-	15-34 U/L
ALT	33	-	47	-	7-40 U/L
CK	297	297	4000	176	46-194 U/L
Aldolase	-	4.3	8.3	-	1.2-7.6 U/L
TSH	1.46	-	-	-	0.55-4.78 uIU/mL
ESR	-	<1	3	-	<=15 mm/hr
CRP	-	0.6	<0.05	-	<1.0 mg/L
Antinuclear antibody screen	-	Negative	Negative	-	Negative
Acetylcholine receptor-modulating antibody	-	0	-	-	0-45%
Acetylcholine receptor-blocking antibody	-	7	-	-	0-26%
Acetylcholine receptor-binding antibody	-	0	-	-	0-0.4 nmol/L
MuSK antibody	-	0	-	-	0
Total lactate dehydrogenase	-	187	189	182	105-230 U/L
Myositis antibody expanded panel^*^	-	Negative	-	-	Negative
Uric acid	-	-	3.7	-	3.3-8.0 mg/dL
HbA1C	-	-	5.3	-	<5.6%
Anaplasma (ehrlichiosis) smear	-	-	Negative	-	Negative
Babesia smear	-	-	Negative	-	Negative
Pompe GAA activity in leukocytes	-	-	-	17.6	5.5-25 nmol/h/mg
Aerobic forearm test	-	-	-	Negative	Negative
Acylcarnitines profile	-	-	-	Normal	Normal
Ammonia	-	-	-	25	7-42 umol/L
Beta-hydroxybutyrate	-	-	-	<0.05	<0.5 mmol/L
Total carnitine	-	-	-	48	30-70 umol/L
Free carnitine	-	-	-	43	23-59 umol/L
Pyruvate kinase, RBC	-	-	-	10.2	4.6-11.2 U/g Hb

In August 2023, he was assessed at a local general neurology clinic, where further testing was performed. This included serum antinuclear antibodies (ANA), a myositis expanded antibody panel, lactate dehydrogenase (LDH) isoenzymes, aldolase, acetylcholine receptor antibody panel, anti-MuSK antibody test, erythrocyte sedimentation rate (ESR), and C-reactive protein (CRP). All these tests returned unremarkable results (Table 1).

In February 2024, several months after his initial consultation, the patient experienced a significant episode characterized by muscle stiffness, pain, and fatigue, which progressively worsened over several days. He reported severe muscle soreness, subjective weakness, and an inability to lift his 33-pound dog in a pool. Additionally, he observed that his urine had become progressively darker despite maintaining adequate hydration. Upon presenting to the emergency department, his CK levels were found to be elevated to 4000 U/L, alongside elevated aldolase levels at 8.3 U/L and mildly elevated aspartate aminotransferase (AST) at 59 U/L and alanine aminotransferase (ALT) at 47 U/L. He was treated with intravenous fluids and required a five-day inpatient stay for monitoring, during which his CK levels gradually normalized to 192 U/L. Further diagnostic tests, including ANA, CRP, ESR, and testing for anaplasmosis and babesiosis, yielded unremarkable results. The nerve conduction study (Table 2) and electromyography (Table 3) were also unremarkable for any myopathy or large-fiber peripheral neuropathy.

**Table 2:** Nerve conduction studies (NCS) in the presented case with muscle fatigue and rhabdomyolysis. AHB: abductor hallucis; EDB: extensor digitorum brevis; NL: normal

Site	Latency (ms)		Amplitude (motor=mV; sensory=µV)		Conduction velocity (m/s)	
	Patient’s value	NL range	Patient’s value	NL range	Patient’s value	NL range
Right peroneal (EDB) motor						
Ankle	3.6	<6.5	5.1	>1.10	-	-
Below the fibular head	9.7	-	5.3	-	50	>36
Lateral popliteal fossa	11.5	-	5.1	-	56	≥42
Right tibial (AHB) motor						
Ankle	6.1	<6.1	16.6	>5.3	-	-
Knee	13.2	-	11.6	-	54	≥42
Right sural sensory						
Calf-lateral malleolus	3.5	<4.5	13	>4	50	≥40
Right superficial peroneal sensory						
Wrist-digit V	3.4	<4.2	13	>5	52	≥40

**Table 3:** Electromyography (EMG) in the presented case with muscle fatigue and rhabdomyolysis. Fasc: fasciculation potentials; FDI: first dorsal interosseous; Fibs: fibrillation potentials; FCR: flexor carpi radialis; NL: normal; Poly: polyphasic; PSW: positive sharp waves; Recrt: recruitment; Spon Disc: spontaneous discharges

Muscle (all right side)	Ins Act	Fibs/ PSW	Fasc	Spon Disc	Amplitude	Duration	Poly	Recrt	Activation
Medial gastrocnemius	NL	NL	NL	NL	NL	NL	NL	NL	NL
Lateral gastrocnemius	NL	NL	NL	NL	NL	NL	NL	NL	NL
Tibialis anterior	NL	NL	NL	NL	NL	NL	NL	NL	NL
Vastus lateralis	NL	NL	NL	NL	NL	NL	NL	NL	NL
Vastus medialis	NL	NL	NL	NL	NL	NL	NL	NL	NL
FDI	NL	NL	NL	NL	NL	NL	NL	NL	NL
FCR	NL	NL	NL	NL	NL	NL	NL	NL	NL
Triceps	NL	NL	NL	NL	NL	NL	NL	NL	NL
Biceps	NL	NL	NL	NL	NL	NL	NL	NL	NL
Deltoid	NL	NL	NL	NL	NL	NL	NL	NL	NL
Mid lumbar paraspinal	NL	NL	NL	NL	-	-	-	-	-
Mid thoracic Paraspinal	NL	NL	NL	NL	-	-	-	-	-

The patient was evaluated in our neuromuscular clinic in March 2024 for further assessment of his hyperCKemia and subjective muscle weakness, stiffness, and myalgias. He reported no relevant medical history, medication use, or family history of neuromuscular conditions, and he denied smoking or drug use. The patient did not also recall any specific triggers for his symptoms, including particular foods or infections (e.g., COVID-19), prior to the onset of his symptoms or before he was later hospitalized. A neurological examination revealed slight asymmetry in scapular muscle bulk but was otherwise unremarkable. Further investigations included tests for Pompe disease (acid alpha-glucosidase, or GAA) enzyme activity, a quantitative acylcarnitine profile, free and total carnitine levels, urine acetone, beta-hydroxybutyrate, and ammonia levels, as well as an aerobic forearm test. All these tests returned unremarkable findings (Table 1).

Following an inconclusive autoimmune and metabolic workup, genetic testing via the Invitae comprehensive neuromuscular disorder panel identified a heterozygous *LPIN1 *exon 18 deletion. There were no other detected mutations in the *LPIN1 *gene, potentially ruling out the possibility of compound heterozygous mutations in this case. The sole variant detected was a gross deletion of the genomic region encompassing exon 18. This out-of-frame deletion is expected to create a premature termination codon, leading to an absent or disrupted protein product. Loss-of-function variants in *LPIN1* are known to be pathogenic [3-6]. As a result, the patient was advised to maintain good fluid intake and to avoid potential triggers such as strenuous exercise, fasting, and febrile illnesses to help prevent episodes of rhabdomyolysis.

## Discussion

This case involves an adult man experiencing recurrent, moderate rhabdomyolysis due to a heterozygous *LPIN1 *exon deletion, with an otherwise unremarkable workup. To our knowledge, this is the first reported case of recurrent rhabdomyolysis in a heterozygous *LPIN1 *adult patient without prior exposure to statins.

The significance of *LPIN1* mutations in severe, recurrent rhabdomyolysis was first identified by Zeharia et al. in 2008 [5]. They reported three pediatric patients, all under the age of six, who experienced episodes of severe rhabdomyolysis despite an otherwise normal workup. Further research by Michot et al. in 2010 highlighted that homozygous or compound heterozygous *LPIN1* mutations were present in 56% of 29 patients under the age of five, with CK levels exceeding 10,000 U/L during rhabdomyolysis episodes [3]. These findings positioned *LPIN1* mutations as the second most common cause of recurrent rhabdomyolysis, following fatty acid oxidation disorders. In a subsequent study conducted in 2012, Michot et al. [4] expanded their cohort to include older patients and those with milder forms of rhabdomyolysis (CK <10,000 U/L). Among the 35 patients studied, 33 were under the age of six at symptom onset, with one eight-year-old and one 42-year-old patient. All patients experienced severe rhabdomyolysis, and 11 out of the 35 patients died during an episode [4].

The most common pathogenic mutation in *LPIN1* is the exon 18 deletion, which accounts for 86% of mutations in Caucasian patients [4]. This deletion impacts amino acids E766-S838 in the C-terminus of the *LPIN1* protein, leading to a complete loss of function [7]. While* LPIN1 *mutations predominantly affect pediatric populations, there have been isolated cases of adult-onset symptoms. These include a 42-year-old patient in the Michot et al. study in 2012 [4] and a 46-year-old patient reported by Minton et al. in 2020 [8], both of whom had homozygous or compound heterozygous mutations. Zeharia et al. [5] also described a heterozygous adult relative with statin-associated myopathy. However, prior to this case, there have been no reported instances of adult-onset recurrent hyperCKemia in heterozygous patients without statin use.

The molecular mechanisms leading to muscle injury in *LPIN1* deficiency are not fully understood. *LPIN1* is a crucial enzyme involved in lipid metabolism and energy homeostasis. To understand how the loss of *LPIN1* activity leads to myocyte injury, investigators have used various mouse and cell culture models [7,9,10]. Notably, fatty liver dystrophy (fld) mice or those with muscle-specific *LPIN1* deletion show a chronic, progressive myopathic phenotype, distinct from the acute or recurrent/episodic syndrome seen in humans [7,9,10]. This myopathy is characterized by myocyte necrosis, regenerative myofibrils, and fibrotic lesions [11]. In *LPIN1*-deficient mice, damaged mitochondria with reduced oxidative capacity accumulate due to impaired mitophagy [7,9]. Interestingly, transgenic muscle-specific overexpression of *LPIN1* can rescue the phenotype of fld mice, indicating a myocyte intrinsic effect [9]. Loss of *LPIN1* in skeletal muscle leads to very low phosphatidate phosphatase (PAP) enzyme activity and accumulation of phosphatidic acid, which impairs autophagy [11]. The mechanistic explanations for impaired autophagy vary by model. For instance, muscle-specific *LPIN1* knockouts exhibit increased muscle diacylglycerol (DAG) content, while fld mice display DAG depletion, affecting muscle lipid content and protein kinase D (PKD) activation [9]. This leads to defective autophagy and may influence myocyte differentiation. In muscle-specific *LPIN1* knockout mice, the PKD mechanism does not apply due to DAG accumulation. However, these mice exhibit signs of lipotoxic and sarcoplasmic reticular stress [10]. Treatment with agents that enhance fat oxidation or chemical chaperones to alleviate stress can mitigate myopathy in these mice [10]. This demonstrates the complex interplay of lipid metabolism in muscle health and disease.

In conclusion, while adult-onset rhabdomyolysis associated with *LPIN1* deficiency is rare, this case underscores the importance of considering it as a differential diagnosis in patients with recurrent rhabdomyolysis, especially when CK levels normalize between episodes and the workup is otherwise unremarkable. With the increasing use of genomic testing, we anticipate that more cases will be identified in the future.

## Conclusions

We reported a rare instance of episodic rhabdomyolysis in an adult patient carrying a heterozygous *LPIN1* gene mutation. This case underscores the necessity of considering *LPIN1 *mutations in the differential diagnosis for adult patients without prior statin therapy or a family history of neuromuscular disorders, who present with such symptoms. Genetic testing is pivotal in confirming the diagnosis when clinical suspicion arises.

Currently, there is no definitive cure for episodic rhabdomyolysis linked to *LPIN1* mutations, characterized by subjective fatigue, muscle weakness, and stiffness. Treatment remains primarily supportive, focusing on adequate hydration and avoiding known triggering factors.
